# Hsp65-Producing *Lactococcocus lactis* Prevents Antigen-Induced Arthritis in Mice

**DOI:** 10.3389/fimmu.2020.562905

**Published:** 2020-09-23

**Authors:** Guilherme Gusmao-Silva, Sarah Leão Fiorini Aguiar, Mariana Camila Gonçalves Miranda, Mauro Andrade Guimarães, Juliana Lima Alves, Angélica Thomaz Vieira, Denise Carmona Cara, Anderson Miyoshi, Vasco Ariston Azevedo, Rafael Pires Oliveira, Ana Maria Caetano Faria

**Affiliations:** ^1^Departamento de Bioquímica e Imunologia, Universidade Federal de Minas Gerais, Belo Horizonte, Brazil; ^2^Departamento de Morfologia, Universidade Federal de Minas Gerais, Belo Horizonte, Brazil; ^3^Departamento de Genética, Evolução e Ecologia, Instituto de Ciências Biológicas, Universidade Federal de Minas Gerais, Belo Horizonte, Brazil; ^4^Instituto Federal do Paraná, Curitiba, Brazil; ^5^Instituto de Investigação em Imunologia, São Paulo, Brazil

**Keywords:** autoimmunity, *Lactococcus lactis*, heat shock proteins, arthritis, probiotic bacteria, oral tolerance

## Abstract

Oral tolerance is the physiological process that enables the immune system to differentiate between harmless dietary and microbiota antigens from pathogen derived antigens. It develops at the mucosal surfaces and can result in local and systemic regulatory and anti-inflammatory effects. Translation of these benefits to the clinical practice faces limitations involving specificity and doses of antigen as well as regimens of feeding. To circumvent these problems, we developed a recombinant Hsp65 delivered by the acid lactic bacteria *Lactococcus lactis* NCDO 2118 directy in the intestinal mucosa. Hsp65 is a ubiquitous protein overexpressed in inflamed tissues and capable of inducing immunoregulatory mechanisms. *L. lactis* has probiotic properties and is commonly and safely used in dairy products. In this study, we showed that continuous delivery of HSP65 in the gut mucosa by *L. lactis* is a potent tolerogenic stimulus inducing regulatory CD4^+^LAP^+^ T cells that prevented collagen-induced and methylated bovine serum albumin-induced arthritis in mice. Clinical and histological signs of arthritis were inhibited as well as levels of inflammatory cytokines such as IL-17 and IFN-γ, serum titers of anti-collagen antibodies and rheumatoid factor. Oral administration of *L. lactis* induced alterations in microbiota composition toward an increased abundance of anaerobic bacteria such as *Bifidobacterium* and *Lactobacillus*. Tolerance to HSP65 and arthritis prevention induced by the recombinant *L. lactis* was associated with increase in IL-10 production by B cells and it was dependent on LAP^+^ T cells, IL-10 and TLR2 signaling. Therefore, HSP65-producing treatment induced effective tolerance and prevented arthritis development suggesting it can be used as a therapeutic tool for autoimmune diseases.

## Introduction

The gastrointestinal mucosa harbors the largest lymphoid tissue in the human body, with the number of lymphoid cells exceeding the cells in other lymphoid organs by orders of magnitude. Antigenic stimulation in the gut is also intense, with daily absorption of 130–190 g of dietary protein and hundred trillion of commensal bacteria from the microbiota (1, 2). Oral tolerance is the physiological process by which the immune system suppresses specific inflammatory immune responses toward innocuous antigens such as microbiota and food proteins, while maintaining protective responses to pathogenic agents. Mechanisms involved in oral tolerance includes regulatory cytokines and regulatory lymphocytes. Mucosal surfaces are populated by a large number and diversity of these regulatory lymphocytes that are able to control local and systemic immune responses (3, 4). Therapeutic applications of oral tolerance for chronic inflammatory diseases have been experimentally demonstrated for decades, but translation to the clinic practice faces limitations, mainly related to identification of the target antigen, feeding protocols and doses of antigen required (5).

Quantity and frequency of the antigen intake are among the several factors that can influence the outcomes of oral tolerance. It has been shown that ingestion of antigen via gavage induces oral tolerance, but this process can be affected by the age and genetic background of the animal (6, 7). On the other hand, a regimen of continuous feeding, in which the antigen is ingested as part of the diet or the drinking water induces a robust suppression that is not affected by these factors (8). However, the amount of purified protein necessary for this approach in humans is a hindrance to its clinical use.

Another limitation of oral tolerance as therapeutic is the identification of the antigen involved in the pathological process. The target antigens involved in the pathogenesis of chronic inflammatory diseases such as inflammatory bowel diseases (IBD), obesity, or atherosclerosis are still elusive (5). Rheumatoid arthritis (RA) is an autoimmune disease that does not have a single antigenic target for autoimmune reaction. An inflammatory reactivity toward extracellular proteins from cartilage has been identified as part of the pathogenesis of the disease in susceptible individuals and experimental arthritis can be induced using collagen or proteoglycans as antigens (9, 10). In addition, the drivers of autoimmune reaction diverge among patients and they cannot be correlated to the severity of the disease. In some cases, individuals present high levels of anti-collagen antibodies years before the onset of any symptom (11).

Some proteins surpass this restriction for they can interact with a vast network of receptors related to their ubiquity. Hsp65 is one of these proteins with the advantage of being an immunodominant antigen able to induce regulatory T cells during thymic development (12). It belongs to the family of HSP60 chaperones, crucial to cellular function and found in all life forms, with strong structural homology among the taxa. Proteins of this family have a remarkable relationship with the immune system, functioning as a signal of cellular activity and indicating processes such as inflammation, hyperplasia, or oxidative stress through receptors of the innate and the adaptative immune system (13).

Regulatory T cells may present different development and mechanisms of action, but they can interact with heat shock proteins. Thymic developed T_*reg*_ cells usually target self-antigens, such as mammalian HSP60, whereas peripherally developed T_*reg*_ are more likely to target exogenous antigens, such as bacterial Hsp65. These chaperones can also interact with innate receptors, such as TLR, in a suppressive and regulatory way (13). Therefore, proteins from the HSP60 family play a major role in regulating inflammation using different receptors to interact with. A regulatory role of these proteins in RA is now very well known. In fact, the regulatory role of HSP60 emerged in studies using different rodent models of arthritis and diabetes (14, 15).

Initial research has proposed Hsp65 as the main precipitating antigen in the pathogenesis of arthritis and diabetes. But when injected with it, instead of developing the disease, animals became refractory to its induction (13, 16–19). The immunoregulatory properties of the HSP60 family are experimentally established in humans and experimental models (20–22). Clinical trials based on its peptides have already been conducted for arthritis (23) (ongoing NCT01123655) and diabetes (24–26). However, none of these trials are based on continuous contact with high quantities of the whole protein through oral mucosa. One major caveat for feeding Hsp60 or Hsp65 to induce oral tolerance in humans is the large amount of purified Hsp65 required.

To circumvent these caveats and explore the full tolerogenic potential of oral HSP60, our group designed a recombinant strain of *Lactococcus lactis* NCDO2118 able to produce and secrete *Mycobacterium leprae* Hsp65 (27). This acid lactic bacterium is generally regarded as safe, commonly used in the dairy industry and has become increasingly common in recombinant technology (28, 29). Treatment with Hsp65 produced by *L. lactis* (Hsp65-Lac) has been shown to be able to prevent the induction of experimental autoimmune encephalomyelitis (EAE), colitis (30, 31), and wild type NCDO2118 strain of *L. lactis* showed beneficial properties in experimental colitis (32).

In this study, we demonstrated that oral treatment with Hsp65-Lac was able to prevent the induction of chronic and acute models of arthritis. The collagen induced chronic model of arthritis (CIA) resembles several features of RA, including induction of anti-collagen antibodies and rheumatoid factor. It is induced by immunization with a self component (type II collagen) mixed to a highly immunogenic protein, ovalbumin (OVA), adsorbed in complete freund adjuvant (CFA) (33, 34). To confirm our results, we also tested an acute model of arthritis induced by methylated bovine serum albumin (mBSA) (35). Although there are several pieces of evidence on the tolerogenic role of Hsp65 in different arthritis models, including ingestion of recombinant *Mycobacterium* Hsp65 (36, 37), the use of recombinant *L. lactis* has advantages: it has anti-inflammatory properties of its own, it survives through the digestive tract and delivers large amounts of Hsp65 directly into the mucosa, boosting its tolerogenic effect (30). In addition, *L. lactis* is a gram-positive lactic bacterium that produces an endotoxin free version of HSP65. Here we show that oral treatment with recombinant HSP65-producing *L. lactis* can also prevent the development of acute and chronic experimental arthritis and that the NCDO2118 strain of *L. lactis* induced some beneficial effects in the microbiota composition.

## Materials and Methods

### *Lactococcus lactis* Strain and Growth Conditions

Two different plasmids were used to transform the *L. lactis* NCDO2118 as described elsewhere (27). The constructed plasmid vector pSEC:hsp65 used a xylose-inducible expression system (XIES) and directed the expression of Hsp65 to the extracellular medium. And an empty plasmid (EP; pXylT:SEC without hsp65) was used to transform *L. lactis* NCDO2118, used as a control. Both vectors included chloramphenicol resistance.

Recombinant *L. lactis* NCDO2118 strains were grown in Difco M17 broth, supplemented with either 0.5% glucose (GM17) or 1% xylose (XM17) and chloramphenicol (10 μg/mL) at 30°C without agitation. On the first day, single colonies of recombinant *L. lactis* harboring an empty plasmid (*L. lactis*-EP) and *L. lactis* NCDO2118 harboring pXylT:SEC:hsp65 (Hsp65-lac) in 5 mL of GM17 were cultured. On the second day, the overnight culture was diluted 1:10,000 in XM17 to induce the expression of the plasmid. On the third day, the animals received the recombinant *L. lactis*, or the *L. lactis*-EP as a control, in XM17 to drink.

Biosecurity and institutional safety procedures were performed in accordance to the standards of *Comissão Técnica Nacional de Biossegurança* (CTNBIO), Brazil, for work with genetically modified microorganisms.

### Animals

Female BALB/c and C57BL/6 mice at six weeks of age were purchased from the Central Animal Facility (CEBIO) of UFMG and treated with ivermectin. TLR2^–\^^–^ C57BL/6 mice were kindly provided by Dr. Sérgio Costa Oliveira, Universidade Federal de Minas Gerais, Belo Horizonte, Brazil, and IL10^–\^^–^ 129 Sv/Ev mice kindly provided by Dr. Donna-Marie McCafferty, University of Calgary, Calgary. Mice were bred and housed in cages maintained in ventilated racks at our experimental animal facility located at *Instituto de Ciências Biológicas, Universidade Federal de Minas Gerais*. All procedures were approved by the local ethical committee for animal research (CEUA-UFMG, Brazil, protocol 118/2013). Experiments were performed in accordance with guidelines and regulation established by the *Conselho Nacional de Controle de Experimentação Animal* (CONCEA), Brazil.

### Experimental Groups

Mice were subjected to the following treatments: N – naive group, control non-manipulated mice; C – positive control group, mice fed only XM17 media before arthritis induction; EP – mice fed recombinant control *L. lactis* bearing an EP before induction of arthritis; HSP – mice fed recombinant *L. lactis* bearing Hsp65-containing vector (Hsp65-lac) before arthritis induction. These groups were used for most experiments (multiple independent ones with *n* = 5–6).

For *in vivo* neutralization of LAP^+^ T cells, mice received three intraperitoneal injections of either anti-LAP monoclonal antibody (20 mg/mouse/day; clone TW716B4) or isotype control (IgG1) on every other day beginning at the first day of *L. lactis* administration. TLR2^–/–^ and IL10^–/–^ mice were used to induce the acute arthritis model after treatment with *L. lactis.*

### Treatment With *Lactococcus lactis*

Oral treatment with the bacteria was performed by offering either XM17 medium (control), XM17 medium containing *L. lactis* EP or Hsp65-producing *L. lactis* at 1 × 10^9^/mL in the drinking water for four consecutive days. Mice consumed an average of 5 mL/day. Bottles were measured for liquid consumption and changed every day. The total dose of bacteria per mouse was estimated to be 5 × 10^9^ CFU and the total daily dose of Hsp65 was about 35 μg per mouse (30).

### Chronic and Acute Models of Arthritis

**FIGURE 1:**
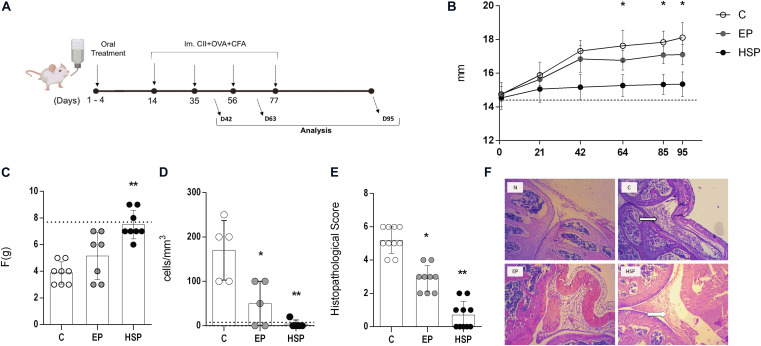
Oral administration of Hsp65-producing *L. lactis* prevented collagen-induced arthritis in mice. **(A)** Schematic protocol for the chronic model of collagen-induced arthritis. BALB/c mice were fed either XM17 broth only (C group), EP-Lac (EP group) or Hsp65-Lac (HSP group) for four days. Ten days later, arthritis was induced with four injections of CII+OVA in CFA; at day 95 all mice were euthanized. **(B)** Average edema of both hind paws was measured with a pachymeter weekly. Graphs are representative of three combined independent experiments (*N* = 16–17). Values from naïve unmanipulated mice are shown as a dashed line **(C)** Mechanical nociceptive threshold was quantified using an Analgesy-meter (Ugo Basile, Italy) at the day 95 (*N* = 8). **(D)** Leukocytes in the synovial cavity were counted after the cavity of the left knee was washed, and the number of total leukocytes (cells/mm^3^) was represented by bars (*N* = 5). **(E)** Histopathological score based on inflammatory infiltrate, pannus, cartilage, and bone erosion was evaluated (*N* = 10). **(F)** Histological sections of the knee collected at day 95 and stained with HE. 100× magnification. The arrow indicates the inflammatory infiltrate in the synovial cavity. Values represent the mean ± SEM. *p < 0.05. **p < 0.005 ***p < 0.0005 in comparison to control group.

Ten days after the end of the treatment with *L. lactis* (day 14 of the experiment), two antigens were used in the chronic model: 2 mg/mL OVA and 2 mg/mL collagen type II chicken (CII) in saline solution was used in the emulsion with an equal volume of CFA plus 4 mg/mL of macerated *Mycobacterium tuberculosis*. After that, every three weeks (days 35, 56, and 77), animals received three subcutaneous injections with 100 μL into the base of the tail as previously described (33, 34). Finally, mice were euthanized on day 95 of the experiment (Figure 1A). Kinetics of the disease was recorded by collecting material a week after each immunization.

For the acute model, C57BL/6 mice received subcutaneous injection in the base of the tail with 100 μL saline solution containing 10 mg/mL mBSA diluted in an equal volume of CFA plus 4 mg/mL *Mycobacterium tuberculosis* H37RA as in the chronic model, 10 days after the treatment with *L. lactis* (day 14 of the experiment). Fourteen days later (day 28), animals were challenged with an injection of 10 μL of a saline solution containing 10 μg mBSA directly into the knee joint as previously described (35). Finally, mice were euthanized on day 29 of the experiment (Figure 5A).

### Measurement of Hind Paw Swelling and Sensitiviy

To evaluate alterations induced by CIA, volume and sensitivity of the hind paws of each animal were measured using both a pachymeter and a plethysmometer (Ugo Basili, Italy) for volume measurement and an analgesy-meter (Ugo Basili, Italy) for sensitivity evaluation. The volume of the two hind paws were taken and the average of values calculated. The sensibility test was performed three times to allow the animals to get used to the procedure. Only the fourth measure was used for evaluation.

### Euthanasia and Sampling

Seven days after the challenge (acute model) or after the immunization (chronic), mice were injected i.p. with Ketamine (60 mg/kg) and xylazine (8.0 mg/kg). When completely sedated, blood was collected through the aorta, sampled and centrifuged (3000 rpm) for 10 minutes after coagulation. Serum was stored in individual tubes and frozen (−20°C). Spleen, mesenteric and inguinal lymph nodes were collected. The right knee of each animal was removed for histology and the left used to obtain the articular material or the synovial wash. Chronic model (CIA) was carried out up to the endpoint (day 95) four times. Lymphoid organs and cells were collected at different time points by euthanizing a group of animals (days 42, 63, and 95 as shown in Figure 1A).

### Synovial Wash

The intra-articular lavage was obtained injecting 10 μL solution of 3% bovine serum albumin (BSA) into the knee joint, collecting the solution and diluting it into 90 μL 3% BSA solution to obtain the lavage fluid. Aliquots of 30 μL joint lavage were diluted in 60 μL of Turk liquid and total white cell counts were obtained in a Neubauer chamber with the aid of optical microscopy (100× magnification).

### Histology

Knee joint specimens were fixed in formalin (10% formaldehyde 37%, 0.65% Na_2_HPO_4_, pH 7.2) for 48 h. Samples were rinsed in running demineralized water solution containing 10% EDTA pH 7.2 at room temperature for a period of three weeks. Pieces were washed in water, dehydrated in alcohol baths, cleared in xylene and embedded in histological paraffin blocks. The 4 μM sections obtained using a microtome were stained with Hematoxylin & Eosin (HE). Slides were qualitatively evaluated using the following parameters: synovial hyperplasia (pannus formation), cell exudate, depletion of cartilage and bone erosion. The severity was scored as: not observed (0), present (1) and intense (2) as previously described (35) as shown in Supplementary Figure S2.

### Measurement of Serum Antibodies

Serum antibodies to OVA, type II collagen, Hsp65, and mBSA were assessed by ELISA. Briefly, 96-well microtiter plates (NUNC) were coated with a solution of OVA (5 μg/μL), CII (5 μg/μL), mBSA (2 μg/μL), or *M. tuberculosis* Hsp65 (1 μg/μL) overnight at 4°C. Plates were incubated with serum samples and bound antibodies detected using alkaline phosphatase conjugated goat anti-mouse IgG (Southern Biotechnology). Color reaction was developed at room temperature with orthophenylenediamine (OPD; 1 mg/mL), 0.04% H_2_O_2_ substrate in sodium citrate buffer. Reaction was interrupted by the addition of 20 μL/well of 2N H_2_SO_4_. Absorbance was measured at 492 nm by an ELISA reader (Bio-Rad Model 450 Microplate Reader). Results were calculated using the running sum of ODs of 6 dilutions starting at 1:100 and ending at 1:102,400 (6 serial 1:4 dilutions). This methodology represents more precisely antibody titers as previously described by our group (38). Alternatively, concentration of IgG1 anti OVA were obtained by interpolating a standard curve obtained with anti Ova IgG1 antibody (monoclonal OVA-14, Sigma). Rheumatoid Factor were measured using a specific kit (MyBioSource).

### Cell Cultures

The organs were isolated macerated and the cells adjusted in concentration 1 × 10^6^ cells/well in RPMI culture (Hyclone, Logan, UT, United States) supplemented with 10% fetal bovine serum (CULTILAB, Campinas, SP, Brazil), 2 mM of l-glutamine (Gibco-BRL, Life Technologies, Grand Island, NY, MO, United States), 25 mM HEPES (Sigma, St. Louis, MO, United States), 50 μM 2-mercaptoethanol (Pharmacia Biotech, Uppsala, Switzerland) and 20 μg/mL gentamicin sulfate (Schering-Plough, Rio de Janeiro). To evaluate production of cytokines, cells were stimulated with 50 μg/mL CII or 10 μg/mL mBSA. A positive control to each sample was stimulated with 1 μg/mL anti-CD3 antibody (Bioscience) and a negative without stimulation. After 48 h in a CO_2_ incubator the supernatants were collected for measurement of IL-17, IFN-γ by ELISA.

### Cytokine Measurement

Cytokines present in the supernatants of the cell cultures were evaluated by sandwich ELISA. Briefly, 96-well polystyrene plates (Nunc) were coated with anti-INF-γ and anti-IL-17 antibodies (BD Pharmingen, San Diego, CA, United States) and incubated for 18 h at 4°C. Plates were washed and 50 μL/well supernatant were incubated for 18 h at 4°C. Detection of cytokines was performed using biotin-conjugated anti-IL-17 and anti-INF-γ followed by incubation with streptavidin-peroxidase. Color reaction was developed at room temperature with orthophenylenediamine (OPD; 1 mg/mL), 0.04% H_2_O_2_ substrate in sodium citrate buffer. Reaction was interrupted by the addition of 20 μL/well of 2 N H_2_SO_4_. Absorbance was measured at 492 nm by an ELISA reader (Bio-Rad Model 450 Microplate Reader).

### Flow Cytometry Analysis

Allophycocyanin-conjugated (APC) mAbs to Foxp3 and CD62L; Phycoerythrin-conjugated (PE) mAbs to CD4, CD44, TNF-α, and LAP; PerCPCy5.5-conjugated mAbs anti-CD4, IFN-γ, and rat IgG2a isotype control from BD Biosciences (San Jose, CA, United States) were used as markers for T cells. PerCP-Cy5.5-CD19, FITC-CD25, and PE-conjugated anti IL-10 from Biolegend (San Diego, CA, United States). For surface antigen detection, cells were labeled with monoclonal antibodies for 30 min at 4°C. For intracellular labeling of cytokines and transcription factors, a fixation/permeabilization kit (e-Bioscience, San Diego, CA, United States) was used after this step. Samples were then incubated for 30 min with a solution containing the appropriate antibodies. After washing with PBS containing 0.5% FBS, samples were fixed with 3% paraformaldehyde for 30 min, washed and stored in PBS at 4°C. Cells were acquired using a FACSCanto II cytometer (Becton Dickinson, East Rutherford, NJ, United States) and data was analyzed by FlowJo software (Tree Star, Ashland, OR, United States). At least 30,000 events were acquired for each analysis. Gating strategies are detailed in Supplementary Material (Supplementary Figure S1).

### Analysis of the Microbiota Profile by Selective Media

Media used for selective bacteria growth were BHI, BBE, MRS Broth, Mackonkey, Agar-Manitol and Agar-Blood. Feces were collected from the entire gastrointestinal tract of the animals and diluted in four concentrations (2×, 4×, 6×, and 8×). Different media were placed in separated Petri dishes, stored in the refrigerator at 4°C until solidified, and then feces were plated. Dishes were incubated for 24 h at 37°C in aerobic and anaerobic conditions, bacterial colonies were counted and analyzed.

### Statistical Analysis

All results were expressed as the mean ± SD of the mean. Significance of differences among groups was determined by either Student’s *t*-test or analysis of variance (ANOVA) with Tukey’s range test. Most of the data represent results from two or three independent experiments with five or six mice per group as indicated in the legend of figures. Means were considered statistically different when *p* < 0.05.

## Results

### Oral Treatment With Hsp65-Producing *Lactococcus lactis* Prevented Chronic Collagen-Induced Arthritis Development

The pathological process triggered by CIA included joint swelling, paw edema and synovial inflammatory infiltrate as observed in the untreated control group (Figure 1B). Oral treatment with Hsp65-lac was able to fully prevent paw swelling (figure 1B) maintaining the volume closer to the one found in naïve mice throughout the experiment. Alternatively to the measurement of hind paw swelling by a pachymeter, we also used a plethysmometer, capable of measuring small changes in volume, in one separate experiment and confirmed that mice from the control group (C) had a high paw swelling whereas Hsp65-Lac-treated mice had a significant reduction in paw volume (Supplementary Figure S2B). Interestingly, hind paw swelling remained unchanged from the day 60 up to day 90 showing that a plateau was reached. Hind paw edema is associated with increased sensitivity to pressure by force. Hsp65-lac treated mice showed also a decreased sensitivity to pressure when compared to control diseased mice (Figure 1C). Synovial wash from untreated mice had a high number of leukocytes, while *L. lactis*-EP treated animals scored less and most animals from group HSP did not show any infiltrating cell in the synovial fluid (Figure 1D). Inflammatory infiltrate, pannus formation and bone degeneration were observed in HE stained sections of control mice and there were absent from Hsp65-lac-treated mice (Figures 1E,F). Oral treatment with Hsp65-lac prevented joint damage, while *L. lactis*-EP had an intermediate effect with decrease in edema and inflammation when compared to control animals.

### Oral Treatment With Hsp65-Producing *L. lactis* Reduced Specific Pathogenic Antibodies

**FIGURE 2:**
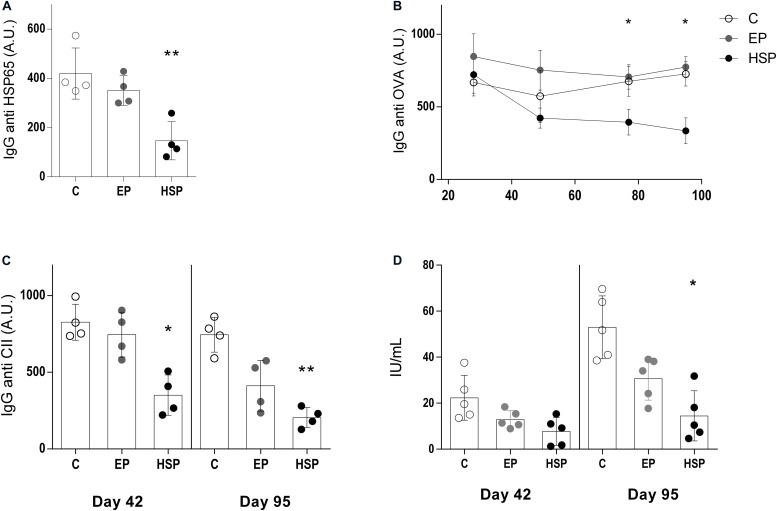
Oral administration of Hsp65-producing *L. lactis* inhibited pathogenic serum antibodies. BALB/c mice were fed either XM17 broth only (C group), *L. lactis*-EP (EP group) or Hsp65-Lac (HSP group) for four days. Non-manipulated naïve animals were used to obtain basal levels (N group, dashed line). Ten days after treatment, arthritis was induced with four injections of CII+OVA in CFA. Sera were collected one week after each booster injection and at the end of the experiment (day 95). Graphs are representative of three independent experiments. **(A)** Levels of serum anti-HSP65 IgG, **(B)** anti-ovalbumin IgG, **(C)** anti-collagen IgG, and **(D)** rheumatoid factor were measured by ELISA. Values represent the mean ± SEM. *p < 0.05 compared to control C group. **p < 0.005 compared to control C group. ***p < 0.0005.

Specific antibodies are of great relevance to RA pathogenesis. Serum antibodies observed during CIA development included high levels of anti-HSP65 (Figure 2A), anti-OVA (Figure 2B), and anti-type-II-collagen IgG (Figure 2C). Rheumatoid factor was also induced by CIA in untreated control mice (Figure 2D). Oral treatment with Hsp65-lac, but not with *L. lactis*-EP, inhibited the production of all these pathogenic antibodies. Hsp65-lac and *L. lactis*-EP reduced the levels of anti-OVA IgG1 in the beginning and at the end of the experiment using a chronic arthritis model (Supplementary Figure S2C).

### Hsp65-Producing *L. lactis* Reduced Inflammatory Cytokine Production During CIA Development

**FIGURE 3:**
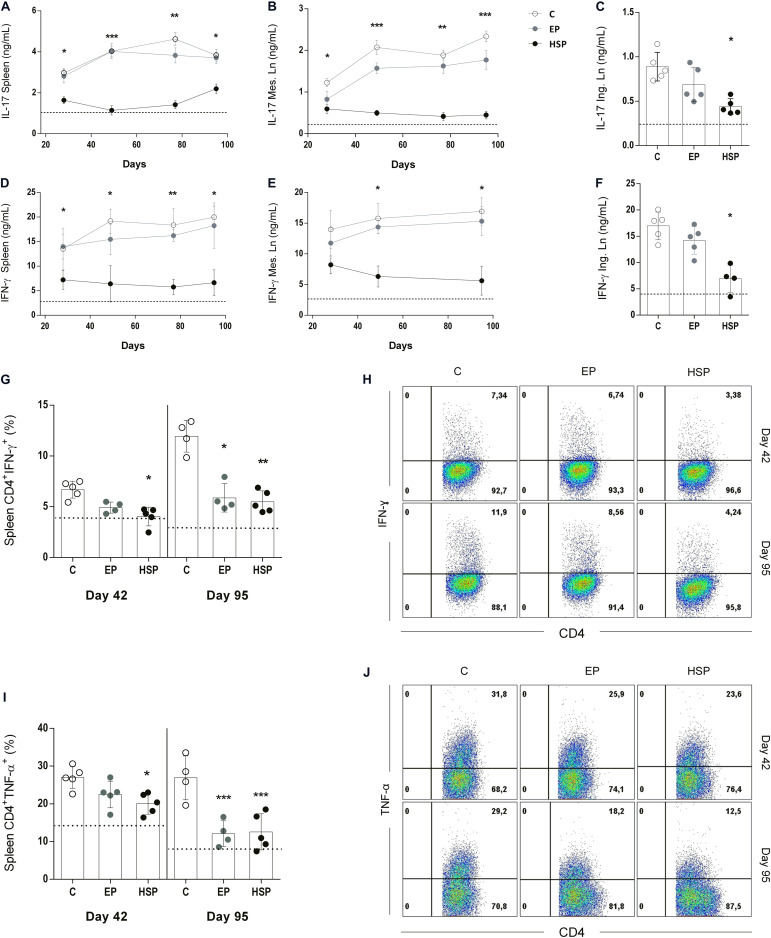
Oral administration of Hsp65-producing *L. lactis* reduced the production of inflammatory cytokines. BALB/c mice were fed either with XM17 broth only (C group), *L. lactis*-EP (EP group) or Hsp65-Lac (HSP group) for four days. Non-manipulated naïve animals were used to obtain basal levels (N group, dashed line). Ten days after treatment, arthritis was induced with four injections of CII+OVA in CFA, as in the CIA protocol. Cytokine production during disease development was assessed by harvesting spleens and mesenteric lymph nodes (mLN) of the animals one week after injection, and inguinal lymph nodes (ILN) on day 95. ELISA assays were used to measure IL17 in cultures of spleen **(A)**, mLN **(B)**, and ILN **(C)** cells; IFN-γ in cultures of spleen **(D)**, mLN **(E)**, and ILN **(F)** cells stimulated with type II collagen (CII). Frequencies of CD4^+^ T cells producing IFN-γ **(G,H)** and TNF-a **(I,J)** in spleens of mice at days 42 (second immunization) and 95 (end of the experiment) were evaluated by intra-cellular staining of the cytokines and analysis by flow cytometry. Gate strategy for analysis of these T cell populations are shown in Supplementary Figure S1. Values represent the mean ± SEM. *p < 0.05 compared to control C group. **p < 0.005 compared to control C group. ***p < 0.0005.

Next, we investigate the production of IFN-γ and IL-17 by cultured spleen cells during CIA development since these two inflammatory cytokines are major inducers of both innate and adaptive immune responses in RA. Hsp65-lac treated mice had lower levels of IL-17 and IFN-γ (Figure 3) in cell cultures of spleen (Figures 3A,D), and mesenteric lymph nodes (Figures 3B,E) stimulated with collagen throughout arthritis development as well as in cultures of inguinal lymph node cells (Figures 3C,F) at day 95. Since effector inflammatory CD4^+^ T lymphocytes are critical in arthritis progression, we also examined the frequencies of IFN-γ^+^CD4^+^ T cells and TNF^+^CD4^+^ T cells at two time points of disease development (day 42 and day 95). Frequencies of both IFN-γ-producing (Figures 3G,H) and TNF-producing (Figures 3I, J) CD4^+^ effector T cells were higher at day 42 and day 95. Oral treatment with Hsp65-producing *L. lactis* reduced the frequency of both effector T cell types at day 42 and 95 when compared to control group. Interestingly, *L. lactis* bearing the EP was also effective in reducing these cells at day 95 suggesting that *L. lactis* by itself mediate some anti-inflammatory action as well. Rise in inflammatory cytokine production by CD4+ T cells was related to a parallel increase in the frequency of effector CD4^+^CD62L^–^CD44^+^ T cells, and this was also prevented by Hsp65-lac oral treatment (Supplementary Figure S3A).

### Oral Treatment With Hsp65-Producing *L. lactis* Increased the Frequency of Regulatory T and B Cells

**FIGURE 4:**
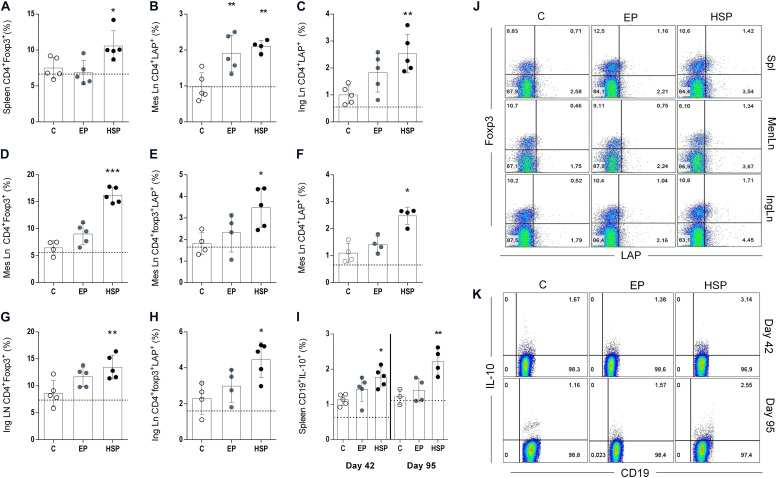
Oral treatment with Hsp65-producing *L. lactis* increased the frequencies of regulatory T lymphocytes. BALB/c mice were fed either XM17 broth only (C group), *L. lactis*-EP (EP group) or Hsp65-Lac (HSP group) for four days. Non-manipulated naïve animals were used to obtain basal levels (N group, dashed line). Ten days after treatment the arthritis was induced with four injections of CII + OVA in CFA, as in the CIA protocol. Spleen, mesenteric and inguinal lymph nodes were collected after the second immunization (day 42) and at day 95 for flow cytometry analysis. Frequencies of CD4+CD25+ from spleen cells collected at day 42 and positive for foxp3 **(A)**; CD4+CD25+ from mesenteric lymph nodes that were LAP+ **(B)**; CD4+CD25+ cells from inguinal lymph nodes obtained at day 42 that were LAP+ **(C)**; CD4+CD25+ mesenteric lymph node cells obtained at the end of the experiment (day 95) that were Foxp3+ **(D)** Foxp3+LAP+ **(E)**, and LAP+ **(F)**. Cells from inguinal lymph nodes collected at day 95 stained for CD4+CD25+Foxp3+ **(G)** and CD4+Foxp3+LAP+ **(H)**. Spleen cells collected after second immunization (day 42) and at day 95 and stained for CD19 and IL-10 **(I)**. Representative plots of cellular populations gated from CD4+CD25+ cells **(J)**. Representative plots of CD19^+^IL10^+^ cells from spleen **(K)**. Values represent the mean ± SEM. *p < 0.05 compared to control C group. **p < 0.005 compared to control C group. ***p < 0.0005.

Many pathological processes are associated with alteration in the frequency of regulatory lymphocytes. Decrease of T_*reg*_ cells have been observed in RA patients, and clinical improvement was also associated with increased or restoration of T_*reg*_ function (39). We have previously shown that oral treatment with Hsp65-producing *L. lactis* resulted in high frequencies of Foxp3^+^CD25^+^CD4^+^ T_*reg*_ cells as well as regulatory CD4^+^ T cells expressing the membrane form of TGF-β (Latency Associated Peptide or LAP) in EAE (30). Attenuation of collagen-induced arthritis with anti-CD3 monoclonal antibody is also associated with induction of LAP^+^ regulatory T cells (40). In the present study, we confirmed that oral treatment with Hsp65-lac previously to CIA induction was associated with high frequencies of Foxp3^+^CD4^+^ T_*reg*_ cells in the spleen (Figures 4A,J) and high frequencies of CD4^+^LAP^+^ cells in mesenteric and inguinal lymph nodes after the second immunization at day 42 (Figures 4B,C,J). At day 95 of CIA development, the augmented frequencies of these cells were observed only in the lymph nodes suggesting that the long-lasting effects of Hsp65-lac were regionalized. Mesenteric lymph nodes (mLN) from Hsp65-lac-treated mice had higher frequencies of CD4^+^Foxp3^+^ (Figures 4D,J), CD4^+^Foxp3^+^LAP^+^ (Figures 4E,J), and CD4^+^LAP^+^ (Figures 4F,J) T_*reg*_ cells than mLN from control mice. In addition, inguinal lymph nodes (iLn) of Hsp65-lac-treated mice had increased frequencies of CD4^+^Foxp3^+^ (Figure 4G) and CD4^+^Foxp3^+^LAP^+^ (Figure 4H) T reg cells than iLN from control mice. B lymphocytes can also exhibit a regulatory function (B_*reg*_ cells), mainly through IL-10 production (41). Decrease in the frequency of this cell population has been reported in rheumatic patients (42). We observed increased frequencies of B_*reg*_ cells (CD19^+^IL10^+^) in spleens of mice treated with Hsp65-lac prior to CIA induction (Figures 4I,K). These findings point to other mechanisms involved in Hsp65-lac, in accordance to the importance reported in the literature for B_*reg*_ in RA.

### Oral Treatment With Hsp65-Producing *L. lactis* Also Prevented Induction of an Acute Model of Arthritis

**FIGURE 5:**
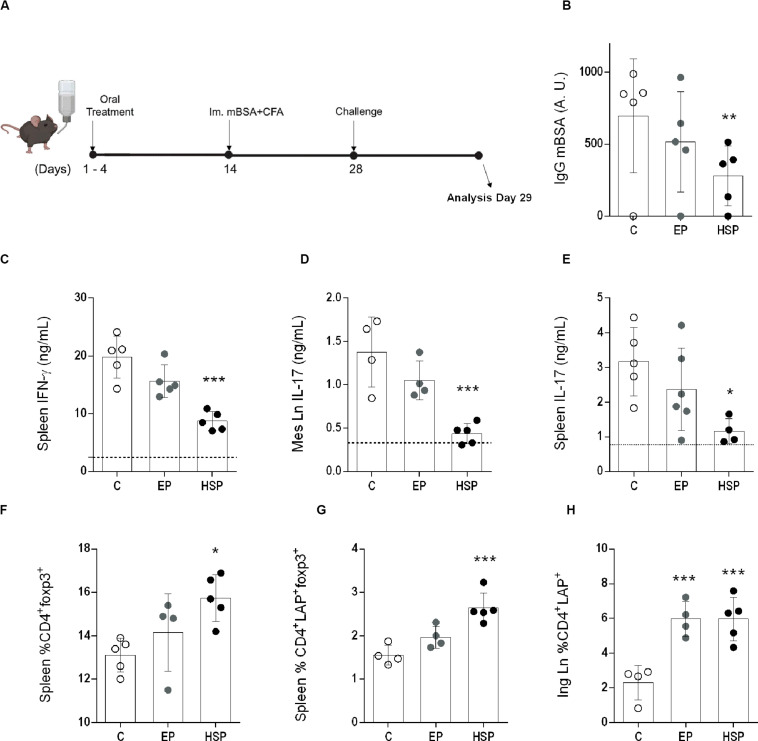
Oral treatment with Hsp65-producing *L. lactis* prevented mBSA induced arthritis (AIA). **(A)** Female C57BL/6 mice were fed either XM17 broth (group C), *L. lactis*-EP (group EP) or Hsp65-Lac (HSP Group) for four days. Ten days afterward, arthritis was induced by a subcutaneous immunization with mBSA in CFA. Two weeks later, mice were injected with mBSA directly into the knee and 24 h after were euthanized. **(B)** Serum anti-mBSA total IgG levels measured by ELISA. **(C)** IFN-γ measured from spleen cell cultures stimulated with mBSA. **(D)** IL-17 from mBSA stimulated cell cultures from mesenteric lymph nodes and **(E)** spleen. **(F)** CD4+CD25+ from Spleen and marked for Foxp3+ and **(G)** Foxp3+LAP+. **(H)** Inguinal lymph node CD4+LAP+. Values represent the mean ± SEM. *p < 0.05 in relation to the control group C. **p < 0.005 compared to the control group C.

The preventive effect of oral treatment with Hsp65-lac was also observed in an acute model of antigen-induced arthritis. The pathogenic process in this model differs from that of the chronic, and Hsp65-lac induced different effects. Specific antibodies were reduced in HSP65-lac treated C57BL/6 mice (Figure 5B), as were the inflammatory cytokines IFN-γ from spleen cells and (Figure 5C) and IL-17 from spleen and mesenteric lymph node cells (Figures 5D,E). Regulatory T cells of different phenotypes (CD4+Foxp3+, CD4+LAP+, and CD4+Foxp3+LAP+) were also elevated by treatment with Hsp65-lac (Figures 5F–H). There was no effect in the frequency of Treg in the mLN as it was found on the chronic model treatment. No difference was identified between mice treated with *L. lactis-*EP and the control untreated group, in contrast with the partial effects of *L.lactis-EP* observed in the CIA. These results suggest that HSP65-producing *L.lactis* may prevent chronic and acute forms of arthritis by triggering different mechanisms of suppression.

### The Tolerogenic Effects of Hsp65-Producing *L. lactis* in CIA Were Dependent on LAP^+^ Cells, IL-10 and TLR2 Signaling

**FIGURE 6:**
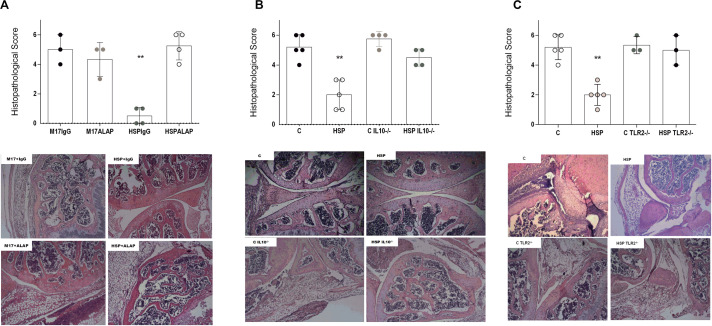
Regulatory components involved in the effect mediated by Hsp65-producing *L. lactis.* BALB/c mice fed Hsp65-lac for four consecutive days were previously injected with Anti-LAP antibody (HSP+ALAP) or IgG4 (HSP+IgG) intermittently for four days. Ten days after treatment arthritis was induced with four injections of OVA+CII in CFA. At day 95 all animals were euthanized, and the right knees were used for preparation of histological sections stained with HE. IL10^–\–^ 129 Sv/Ev and TLR2^–\–^ C57BL/6 were treated with either XM17 broth (C IL10^–\–^ and C TLR2^–\–^) or Hsp65-lac (HSP IL10^–\–^ and HSP TLR2^–\–^). Wild type (WT) mice were used as control for both experiments using the mBSA-induced acute model of arthritis. Histopathological score and HE stained section of **(A)** mice treated with anti-LAP antibody. **(B)** Deficient for IL-10 and **(C)** TLR2. Bars represent the score based on inflammatory infiltrate, pannus, cartilage and bone erosion. Values represent the mean ± SEM. *p < 0.05 compared to control C group. **p < 0.005 compared to control C group. ***p < 0.0005.

Since LAP^+^ T cells have been previously reported as regulatory cells involved in oral tolerance induced by Hsp65-lac (30), we investigated its role on CIA model by blocking the action of these cells with anti-LAP antibodies *in vivo*. BALB/c mice were treated with either XM17 or Hsp65-lac prior to CIA induction, but they were also injected with either anti-LAP or control IgG1 antibodies at intermittent days during oral treatment. Only the HSP65-lac group injected with control IgG1 were protected from the CIA (Figure 6A). Mice treated with Hsp65-lac and injected with anti-LAP antibodies develop CIA with similar severity, measured by histological score, as mice treated with XM17. Considering that we observed an increase in IL-10 producing B_*reg*_ cells (Figure 4I), we also decided to investigate the role of IL-10 in oral tolerance in our model. To address that, acute arthritis was induced in IL10^–/–^ 129Sv/Ev mice after treatment with Hsp65-Lac. Histopathological score demonstrated that this cytokine was also fundamental for the inhibitory effect of Hsp65-lac effect in arthritis development (Figure 6B). Our previous study using HSP65-producing *L. lactis* in a mouse model of colitis showed that its tolerogenic and anti-inflammatory effects were dependent on signaling mediated by TLR2 (32). We confirmed a similar relevance for TLR2 for the effect of Hsp65-Lac in the prevention of the acute model of arthritis, since TLR2^–/–^ mice were not affected by Hsp65-lac treatment (Figure 6C).

### Treatment With *L. lactis* NCDO2118 Strain Resulted in Changes in the Microbiota Composition

**FIGURE 7:**
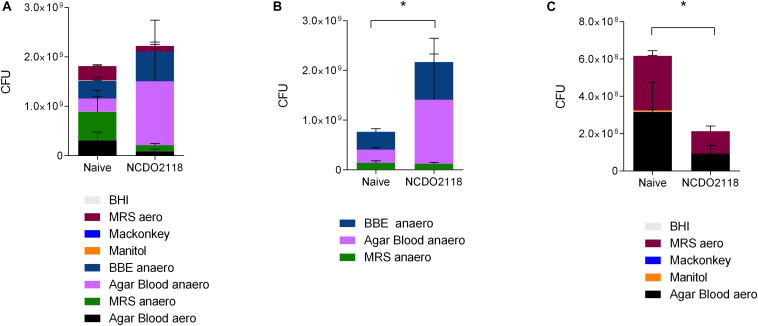
Profile of the small intestine microbiota after *L. lactis* NCDO2118 administration. C57BL/6 mice received *L. lactis* NCDO2118 in the drinking bottle for 4 days. After euthanasia, the entire fecal content of the small intestine was collected and cultured in different media. Representative graphs showing the CFU grown either in all media **(A)**, in media maintained in anaerobiosis **(B)**, or in aerobiosis **(C)** for 24 h. Naive non-manipulated mice were used as control. Statistical significance of the difference between groups was calculated using the ANOVA test. Values represent the mean ± SEM. *p < 0.05.

*Lactococcus lactis* NCDO2118 had been shown to present anti-inflammatory effects when used concomitantly to colitis development (32). We also observed some effect of *L. lactis* EP in the reduction of effector inflammatory cells in CIA model suggesting that *L. lactis* would have an effect *per se* in disease development. Since *L. lactis* is a lactic bacterium that is lodged temporarily in the upper part of the small intestine, we hypothesized that it could interfere with oral tolerance induced in the gut mucosa by altering the microbiota composition. Indeed, we observed that wild type *L. lactis* NCDO2118 induced changes in the profile of the microbiota identified by the growth of fecal contents collected from treated mice in selective media (Figure 7A) under aerobic and anaerobic conditions. Treatment with *L. lactis* NCDO2118 was associated with a decrease in anaerobic bacteria (Figure 7B) and a concomitant increase in aerobic bacteria (Figure 7C).

## Discussion

Our results indicate that oral treatment with Hsp65-producing *L. lactis* prevented the development of collagen-induced and mBSA-induced arthritis in mice inhibiting clinical and histological signs of disease. This effect was related to tolerogenic mechanisms triggered in the gut by feeding Hsp65. Interestingly, tolerance to Hsp65, shown by the reduction in anti-Hsp65 IgG, spread to the antigens used for CIA induction: collagen and OVA. We observed a similar effect of Hsp65-lac in the prevention of the EAE model (30). Antibodies to both antigens were significantly reduced by HSP65-lac. It is noteworthy that rheumatoid factor was also inhibited by HSP65-lac treatment. Production of pathogenic antibodies such as the RF is quite relevant to the development of RA (10), and its inhibition is another piece of evidence for the large spectrum of tolerance achieved by Hsp65-lac treatment in arthritis. The chronic model of arthritis (CIA) was induced and sustained by administration of four successive challenges. Remarkably, none of these challenges were capable of breaking oral tolerance in HSP65-lac-fed mice suggesting that immunoregulatory mechanisms triggered by oral Hsp65-lac were robust enough to sustain tolerance even after repeated inflammatory insults.

The profile of regulatory T lymphocytes induced by Hsp65-Lac treatment was also close to the one observed in a previous study using Hsp65 producing *L. lactis* to prevent EAE (30). CD4^+^LAP^+^ T_*regs*_ are TGF-β producing cells that express the surface form of this cytokine. These T_*regs*_ are fundamental for the development of oral tolerance in different animal models of disease (44). Hsp65-lac treatment induced the increase in the frequencies of CD4^+^CD25^+^Foxp3^+^ and CD4^+^LAP^+^ T_*regs*_. *In vivo* treatment with anti-LAP antibodies clearly demonstrated that LAP^+^ cells were critical for the suppressive effect of Hsp65-lac. Even if the mechanism involved in the prevention of acute and chronic model were different, LAP^+^ T_*reg*_ cells was relevant for the inhibition of both arthritis models.

Oral therapy with Hsp65-lac also increased the frequency of IL-10 producing B cells. It is not yet established the exact relationship between regulatory B cells and production of pathogenic antibodies. Immunization with *M. leprae* DNA-Hsp65 induced a B-cell subpopulation expressing IL-10 (45). These B regulatory cells (B_*reg*_) were inversely associated with symptoms in rheumatic patients (42). Transfer of IL-10^+^ B cells prevented the induction of CIA in mice (41), and these cells were crucial for the suppression of Th17/Th1 responses and induction of T_*reg*_ (46). Treatment with Hsp65-lac increased this the frequency of this cell type in mice during chronic arthritis.

The therapeutic potential of bacterial HSP65 and endogenous HSP60 in either rheumatoid or experimental arthritis has been investigated for decades. However, most treatments are based on Hsp65 derived peptides parenterally delivered. In addition, acute and adjuvant-induced models reproduce only the inflammatory aspects of arthritis, with autoimmune parameters often undetectable. We tested the effects of oral treatment with Hsp65-Lac in mBSA-induced arthritis, an acute model of disease and showed a similar inhibitory effect with reduction in anti-mBSA IgG, inflammatory cytokines (IFN-γ and IL-17), and increased frequencies of CD4^+^Foxp3^+^ as well as CD4^+^LAP^+^ T_*reg*_ (Figure 5) indicating the Hsp65-lac has suppressive effects in arthritis development regardless of their mechanism of inflammation or disease duration.

Studies conducted by our group showed that oral tolerance induced by Hsp65-lac were not directly related to cross reaction with bacterial products present in the adjuvant (30), and that oral treatment with Hsp65-Lac triggered a tolerogenic effect toward antigens used for disease induction. This suggests that Hsp65-lac induces oral tolerance with an indirect effect in related antigens present at the target tissue as described previously for other inflammatory conditions such as wound and granuloma formation (47, 48). *L. lactis* NCDO 2118 strain has been shown to have anti-inflammatory effects in the gut mucosa (31), also a major advantage of using this strain of probiotic bacterium as a delivery system.

One of the main features of immunological tolerance over conventional therapies is being specific and, therefore, avoiding generalized immunosuppression. Treatment with HSP65-lac does not induce suppression or impairment of protective immune responses against infectious agents (30). Other studies have shown the same for HSP60. A clinical trial applying peptides derived from HSP60 in rheumatic patients did not result in immunosuppression. Treated individuals presented higher frequencies of Foxp3^+^CD4^+^ T cells and higher expression levels IL-10 than the placebo group, and none of them presented compromised immune response to tetanus toxin (23). Furthermore, reactivity to HSP60 can be related to protection against several different infectious agents (49–51).

The use of anti-inflammatory drugs along with immunotherapies is not uncommon. The decrease in the inflammatory process, if not essential, provide better performance in these therapies. The downside of this approach is that the process of tolerance derives directly from active immunosuppression, and most of anti-inflammatory drugs interfere with regulatory T cell function. On the other hand, the anti-inflammatory properties of probiotics are mediated by active regulatory mechanisms rather than immunosuppression, offering a possible booster for oral therapy. In our study, although oral treatment with control *L. lactis* (EP) had no effect in arthritis development, its administration resulted in reduced frequencies of effector inflammatory CD4^+^ T cells expressing IFN-γ and TNF-α. Therefore, *L. lactis* NCDO2118 had some anti-inflammatory effects and it may be useful not only for immunotherapy but also for the management of arthritis. Indeed, oral treatment with *L. lactis* NCDO2118 induced a shift in the microbiota composition toward increased abundance of anaerobic microbiota that contains beneficial bacteria such as acid-lactic Bifidobacterium and Lactobacillus. This microbial profile was shown to attenuate colitis in mice (52).

Microbiota manipulation can be relevant for RA pathology and scientific studies showing beneficial effects of probiotics in arthritis have accumulated (53, 54). In a model of collagen-induced arthritis, oral tolerance to collagen was enhanced by its administration along with the probiotic *L. casei*, which by itself can be beneficial (55–59).

The effect of Hsp65-lac was shown to be dependent on TLR-2 signaling in colitis (32) as it was in our model. This can be related to the versatile nature of HSPs, being able to induce either immunization or immunoregulation via interaction with both innate and adaptative receptors. The regulatory effects of HSP60, for instance, were described to be dependent on TLR-9, TLR-4 and Myd88 in different experimental conditions and disease models (60–62).

*L. lactis* is a conventional and commonly used microorganism in the dairy products and it is increasingly studied in recombinant biotechnology as a safe. Efficient to produce exogenous proteins, this microorganism is already one of the most consumed by humans. In this study, we showed that the recombinant strain of Hsp65-producing *L. lactis* NCDO2118 prevented different models of arthritis by inducing different regulatory mechanisms. Human arthritis also presents a multitude of pathogenic processes and demands different and diverse therapeutic approaches.

Our results demonstrated that Hsp65-producing *L. lactis* can boost tolerogenic mechanisms triggered by the oral contact with a highly immunogenic antigen. It was able to prevent pathogenic inflammatory events without the need of immune suppression. Hsp65-producing *L. lactis* in the present format is still not suitable for human use because of the antibiotic resistant gene and the requirement for xylose for Hsp65 induction. However, novel recombinant technologies that makes possible the expression of exogenous proteins in probiotics used for dairy products without the use of antibiotics and exogenous inducing agents are available (62–66). A version of Hsp65-producing *L. lactis* with such features would be suitable for clinical studies. For its ubiquity, Hsp65 is a safe protein for human consumption and our data support the idea that Hsp65-lac can be a useful and safe therapeutic approach to regulate inflammatory process of different origins.

## Data Availability Statement

The raw data supporting the conclusions of this article will be made available by the authors, without undue reservation, to any qualified researcher.

## Ethics Statement

The animal study was reviewed and approved by Comitê de Ética no Uso de Animais – UFMG.

## Author Contributions

GG-S performed the experiments, analyzed the results, and wrote the manuscript. SA performed the experiments to test the role of LAP^+^ cells and TLR2 signaling. MM helped with all flow cytometry analysis, helped the discussion, and the writing of the manuscript. MG helped with the mBSA-induced arthritis experiments. JA performed the microbiota analysis. AV supervised the microbiota analysis. AM constructed the recombinant *L. lactis*. VA supervised the construction of the recombinant *L. lactis*. RO co-supervised the experiments, discussed the results, and helped writing the manuscript. AF designed the study, supervised the experiments, discussed the results, and wrote the manuscript. All authors contributed to the article and approved the submitted version.

## Conflict of Interest

The authors declare that the research was conducted in the absence of any commercial or financial relationships that could be construed as a potential conflict of interest.

## Supplementary Material

FIGURE S1 Strategy for flow cytometry analysis of cytokine-producing cells from BALB/c mice with collagen-induced arthritis (CIA). Cells were harvested from spleens of BALB/c mice treated with XM17 broth (C), *L.lactis*-EP (EP) or HSP-Lac (HSP) for four days and immunized 10 days later with CII + OVA + CFA for CIA induction. IFN-γ-producing (G) and TNF-α-producing (H) CD4^+^ T cells in spleens of mice at days 42 and 95 of disease development were evaluated by intra-cellular staining of the cytokines and analysis by flow cytometry. Gate strategy used to analyze cytokine-producing CD4 ^+^ T cells is shown.

FIGURE S2 Histological, clinical and antibody analysis of in BALB/c mice with collagen-induced arthritis (CIA) treated with HSP65-producing *L.lactis.* Representative histological sections as it was used for score determination **(A)**. Upper row shows intense (1) hyperplasia (pannus), (2) bonne erosion and (3) inflammatory infiltrate. Lower row shows a moderate degree of these same pathological events. For histological scoring purposes, intense events scored 2, moderate 1 and absent, 0. **(B)** Volume of hind paw taken with a plethysmometer. **(C)** Serum anti-OVA IgG1 measured by ELISA. **(D)** Hind paw swelling during chronic arthritis (CIA) development measured by a plethysmometer. **(E)** Anti-OVA IgG1 of BALB/c mice at days 40 and 60 0f chronic arthritis (CIA) development. Values represent the mean ± SEM. ^∗^*p* < 0.05 compared to control C group. ^∗∗^*p* < 0.005 compared to control C group. ^∗∗∗^*p* < 0.0005.

FIGURE S3 Effector CD4^+^ T cells in mice with either chronic arthritis (CIA) or acute arthritis (mBSA-induced) treated with HSP65-producing *L.lactis.* Activation of naive CD4+ T cell into effector cell was analyzed by measuring the frequency of CD4+CD44+CD62L-cells in spleen **(A)** of BALB/c with CIA at days 42 and 95 after disease induction using flow cytometry. BALB/c mice were fed either XM17 broth only (C group), *L. lactis*-EP (EP group) or Hsp65-Lac (HSP group) for four days and immunized for arthritis (CIA) induction 10 days thereafter. Frequencies of spleen **(B)** and mesenteric lymph nodes **(C)** activated CD4+CD44+CD62L-cells of C57BL/6 mice with mBSA-induced arthritis were also analyzed by flow cytometry. C57BL/6 mice were fed either XM17 broth only (C group), *L. lactis*-EP (EP group) or Hsp65-Lac (HSP group) for four days. Ten days afterward, arthritis was induced by a subcutaneous immunization with mBSA in CFA. Two weeks later, mice were injected with mBSA directly into the knee and 24 h after they were euthanized. Graphs show the frequency of CD4-gated CD44+CD62L-cells. Values represent the mean ± SEM. ^∗^*p* < 0.05 compared to control C group. ^∗∗^*p* < 0.005 compared to control C group. ^∗∗∗^*p* < 0.0005.

## Abbreviations

AIA: antigen induced arthritis
CIA: collagen induced arthritis
CII: type II collagen
HSP: heat shock protein
Hsp65-lac: recombinant *Lactococcus lactis* secreting *Mycobacterium leprae* Hsp65
LAP: latency associated peptide
L. lactis-EP: *Lactococcus lactis* bearing an empty plasmid
mBSA: methylated bovine serum albumin
OVA: ovalbumin
RA: rheumatoid arthritis.
