# Supplementary figures and images for: Hsp65-Producing Lactococcocus lactis Prevents Antigen-Induced Arthritis in Mice
Source: Front Immunol. 2020 Sep 23;11:562905. doi: 10.3389/fimmu.2020.562905 (PMC7538670; doi:10.3389/fimmu.2020.562905)

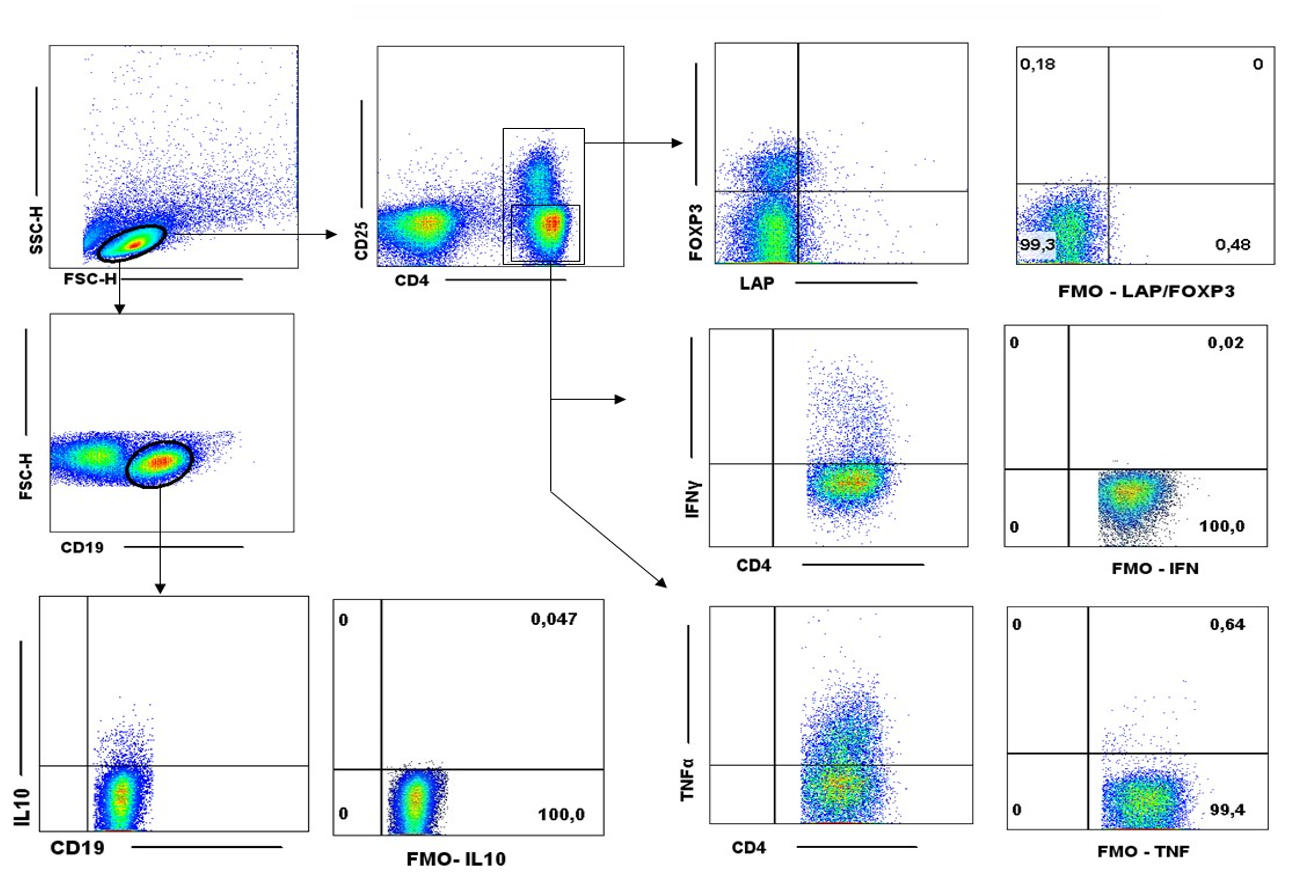

Supplement: FIGURE S1 — Strategy for flow cytometry analysis of cytokine-producing cells from BALB/c mice with collagen-induced arthritis (CIA). Cells were harvested from spleens of BALB/c mice treated with XM17 broth (C), L.lactis-EP (EP) or HSP-Lac (HSP) for four days and immunized 10 days later with CII + OVA + CFA for CIA induction. IFN-γ-producing (G) and TNF-α-producing (H) CD4+ T cells in spleens of mice at days 42 and 95 of disease development were evaluated by intra-cellular staining of the cytokines and analysis by flow cytometry. Gate strategy used to analyze cytokine-producing CD4 + T cells is shown. [file Image_1.tif]

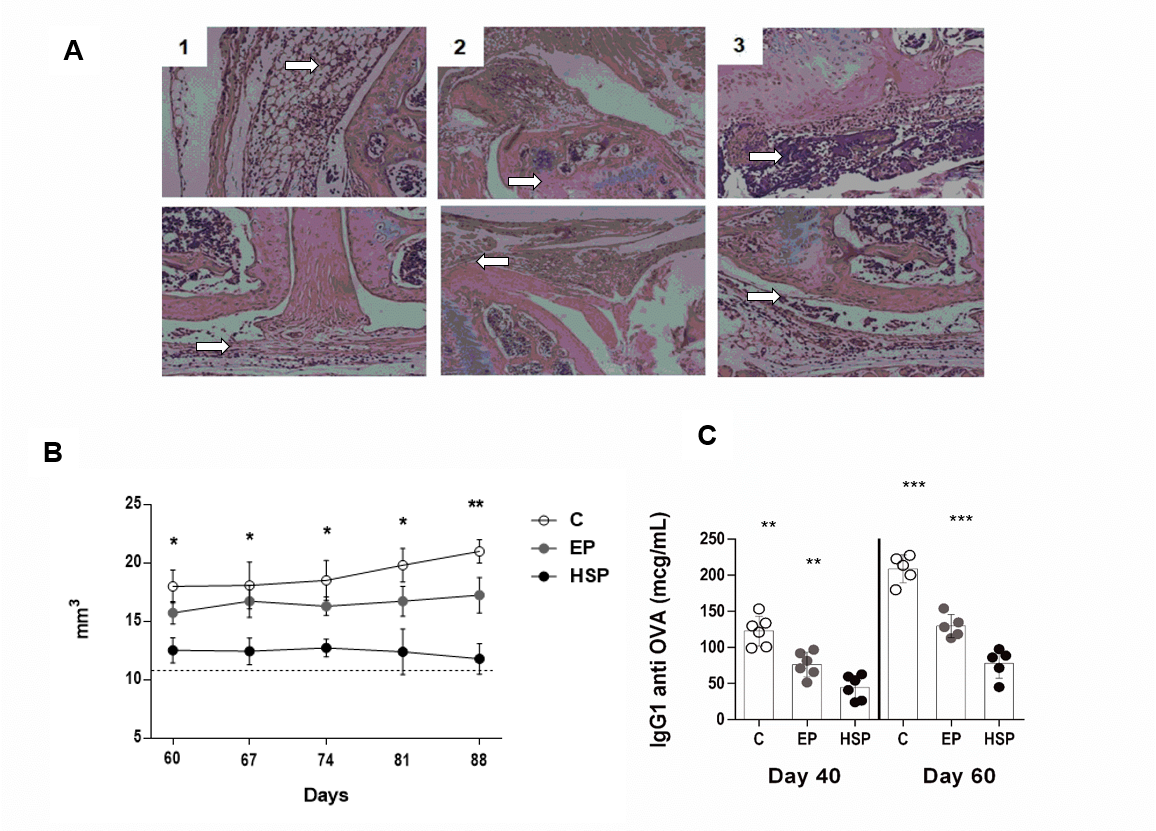

Supplement: FIGURE S2 — Histological, clinical and antibody analysis of in BALB/c mice with collagen-induced arthritis (CIA) treated with HSP65-producing L.lactis. Representative histological sections as it was used for score determination (A). Upper row shows intense (1) hyperplasia (pannus), (2) bonne erosion and (3) inflammatory infiltrate. Lower row shows a moderate degree of these same pathological events. For histological scoring purposes, intense events scored 2, moderate 1 and absent, 0. (B) Volume of hind paw taken with a plethysmometer. (C) Serum anti-OVA IgG1 measured by ELISA. (D) Hind paw swelling during chronic arthritis (CIA) development measured by a plethysmometer. (E) Anti-OVA IgG1 of BALB/c mice at days 40 and 60 0f chronic arthritis (CIA) development. Values represent the mean ± SEM. ∗p < 0.05 compared to control C group. ∗∗p < 0.005 compared to control C group. ∗∗∗p < 0.0005. [file Image_2.tif]
